# Effectiveness of tranexamic acid in burn patients undergoing surgery – a systematic review and meta-analysis

**DOI:** 10.1186/s12871-024-02471-3

**Published:** 2024-03-04

**Authors:** Joeri Slob, Rolf K. Gigengack, Margriet E. van Baar, Stephan A. Loer, Seppe S. H. A. Koopman, Cornelis H. van der Vlies

**Affiliations:** 1 Burn Center Rotterdam, Maasstadweg 21, Rotterdam, 3079 DZ The Netherlands; 2 Department of Intensive Care, Amsterdam UMC, location VU Medical Center, Amsterdam, The Netherlands; 3 Department of Anaesthesiology, Amsterdam UMC, Location VU Medical Center, Amsterdam, The Netherlands; 4 Association of Dutch Burn Centers, Maasstad Hospital, Rotterdam, The Netherlands; 5 Department of Anaesthesiology, Maasstad Hospital, Rotterdam, The Netherlands; 6 Department of Surgery, Maasstad Hospital, Rotterdam, The Netherlands; 7 Department of Surgery, Trauma Research Unit, Erasmus MC, University Medical Centre Rotterdam, Rotterdam, The Netherlands; 8 Department of Public Health, Erasmus MC, University Medical Centre Rotterdam, Rotterdam, The Netherlands

**Keywords:** Burns, Tranexamic Acid, Haemorrhage

## Abstract

**Background:**

Reducing blood loss during excisional surgery in burn patients remains a challenge. Tranexamic acid during surgery can potentially reduce blood loss. The use of tranexamic acid during excisional surgery in burn patients has recently been described in a review and meta-analysis. However, quality assessment on studies included was not performed and this review did not apply independent reviewers. Quality assessment of studies investigating the effectiveness of tranexamic acid in burn patients is crucial before concusions can be drawn. Therefore, we conducted a systematic review and meta-analysis of the literature investigating the effectiveness of tranexamic acid in burn patients undergoing surgery.

**Methods:**

A systematic review and meta-analysis of the literature was conducted. The study was pre-registered in PROSPERO database (CRD42023396183).

**Results:**

Five studies including two randomised controlled trials (RCTs) with a total of 303 patients were included. Risk of bias of the included studies was moderate to high. Individual results of the studies were heterogeneous. In three studies of moderate quality the administration of tranexamic acid resulted in a reduction of blood loss per unit excised area, accounting as moderate level of evidence. In two low-quality studies and one moderate quality study the administration of tranexamic acid resulted in a reduction of transfused packed Red Blood Cells (pRBC’s), accounting for moderate level of evidence. Postoperative haemoglobin levels were higher after tranexamic acid administration in one study, accounting for insufficient evidence. Meta-analysis pooling overall blood loss from two separate RCTs failed to detect a statistically significant reduction. Substantial heterogeneity was observed.

**Conclusions:**

Moderate level of evidence indicates that tranexamic acid reduces blood loss per unit of excised area and transfusion of packed Red Blood Cells. Results indicate that tranexamic acid can be beneficial in burn patients undergoing surgery. More high-quality research is needed to confirm these results. Future studies should focus on the dosing of tranexamic acid, the administration approaches, and even consider combining these approaches.

**Trial Registration:**

PROSPERO: CRD42023396183

**Supplementary Information:**

The online version contains supplementary material available at 10.1186/s12871-024-02471-3.

## Introduction

Blood loss during excisional surgery in burn patients remains a challenge and has been identified as an independent predictor of mortality [1]. Several strategies have been described in literature to reduce blood loss during surgery (i.e., topical adrenaline, tourniquets and tranexamic acid) [2]. Despite the use of these techniques, blood loss remains substantial [3, 4]. This leads to allogenic blood transfusions with all associated risks and costs [5]. Autologous blood transfusion by recovering erythrocytes during burn excisional surgery using a cell saver is possible. However, bacterial contamination in collected erythrocytes makes re-administration in burn excisional surgery not yet feasible [6]. Until use of autologous blood transfusion is deemed feasible, reducing blood loss is the only way to reduce allogenic blood transfusions.

Tranexamic acid is a synthetic lysine analogue that competitively inhibits the activation of plasminogen, thereby inhibiting the degradation of fibrin [7]. This slows its conversion to plasmin at the end of the clotting cascade [7]. Tranexamic acid has a proven efficacy in reducing blood loss in different patient categories [8, 9]. A meta-analysis published in 2021 included 57 studies divided into surgical subspecialties (orthopaedics, obstetrics and gynaecology, oral maxillofacial surgery/otolaryngology, cardiac surgery, and plastic surgery [10]. The perioperative estimated blood loss was lower in patients receiving tranexamic acid [10]. There was no difference in the incidence of venous thromboembolic events between tranexamic acid and control groups [10]. Though proven in other patient categories, the use of tranexamic acid during excisional surgery in burn patients has not been systematically examined yet.

Several observational studies investigated the use of tranexamic acid and its effectiveness regarding transfusion requirements in burn excisional surgery [11, 12]. Moreover, three randomised controlled trials (RCTs) were performed [13–15]. The results indicated that tranexamic acid reduced the intraoperative blood loss significantly. Although Fijany et al. recently published a review and meta-analysis which included above-mentioned studies [16]. The authors pooled results from RCTs with a non-randomised cohort study in the meta-analysis [16]. This decision can be debated as the level of evidence of these two designs differ and thus should not be combined. In addition, the authors did not perform a quality assessment on the studies included. Assessing the methodological quality of evidence is a crucial part of a systematic review before conclusions can be drawn. To enhance generalizability, a summary and quality assessment of available literature is needed which can guide physicians regarding the use of tranexamic acid in burn patients during surgery. Therefore, we performed a systematic review and meta-analysis of available literature. The objective of the study was to gather studies which evaluated the effectiveness of tranexamic acid in burn patients undergoing surgery.

## Material and methods

### Design

A systematic review and meta-analysis of the literature was conducted and reported according to the preferred reporting items for systematic reviews and meta-analysis (PRISMA) guidelines [17]. This study was registered in PROSPERO database (CRD42023396183), before the search was conducted [18]. The study protocol and versions can be found in PROSPERO with above-mentioned registration number.

### Types of studies

The review included RCTs involving at least one intervention group and one control group in which the groups were selected by a random process, or prospective studies or retrospective (database) studies comparing intervention and control groups.

### Types of participants

Burn patients undergoing surgery with any percentage total body surface area (%TBSA) burned and any age were included.

### Types of interventions

The intervention considered was tranexamic acid, regardless the route of administration. Articles investigating the effectiveness of anti-fibrinolytic agents other than tranexamic acid were excluded. Any comparator intervention was accepted.

### Types of outcomes

Articles had to report on at least one of the following outcomes: measuring blood loss intraoperatively (primary outcome), blood loss up to 48 h postoperatively, transfusion requirements, haemoglobin levels or complications/side effects.

### Source information

The systematic literature search was performed in Medline, Embase, Web of Science and Cochrane Central on the 23th of January 2023.

### Search strategy

Tailored search strings were developed by an information specialist with significant experience in conducting systematic reviews from the Erasmus MC Medical Library. The search strings used per electronic database can be found in Appendix 1.

## Data collection

### Study selection

Duplicates were removed from the initial selection of articles. Two review authors (JS and RG) independently examined the titles and abstract of articles to determine which selection was appropriate for further analysis. From this selection full text articles were obtained. These full text articles were read by JS and RG independently. The reviewing authors discussed their selection until consensus was reached. Whenever applicable a third reviewer (SK) arbitrated.

### Data extraction

The following data were gathered from articles included: first author, publication year, study design, sample size, mean %TBSA, intervention/control group specification. For the outcome measures the authors targeted on blood loss, transfused packed Red Blood Cells (pRBC’s) and Haemoglobin. Therefore, three overarching outcomes were formulated: outcome A blood loss (primary outcome), outcome B transfused pRBC’s and outcome C Haemoglobin level. These outcomes were extracted from the original articles.

### Methodological quality

The quality assessment of articles included was carried out by JS and RG independently. Whenever applicable a third reviewer (SK) arbitrated. The revised Cochrane risk of bias 2 tool for randomised controlled trials (RoB 2) was used for included randomised controlled trials [19]. This tool uses stratification into five domains to detect potential bias. For included articles other than randomised controlled trials the risk of bias tool to assess non-randomised studies of interventions (ROBINS-I) was used [20]. This tool uses stratification into seven domains to detect potential bias. Based on the comments within the domains’ signalling questions concerning the RoB2 and ROBINS-I, a finalized risk of bias judgement is formed. Low risk of bias if all the domains are judged as ‘low risk of bias’. Some concern if ‘some concern’ in at least one domain is judged but without a ‘high risk of bias’ in any domain. High risk of bias is given if ‘high risk of bias’ is judged in at least one domain or the study is judged to have ‘some concern’ for multiple domains in a way that substantially lowers confidence in the result [19, 20]. Quality assessment was conducted for each pre-specified outcome (outcome A,B,C).

### Synthesis

A narrative summary of studies included is presented. A best-evidence synthesis was applied following Proper et al. Three levels of evidence are distinguished 1) strong evidence: consistent findings in multiple high-quality studies (if there are two or more high-quality studies, the studies of low methodologic quality are disregarded) 2) moderate level of evidence: consistent findings in one high quality study and at least one low-quality study, or consistent findings in multiple low-quality studies 3) insufficient level of evidence: only one study available or inconsistent findings in multiple studies [21]. Best evidence synthesis results were considered consistent when at least 75% of the studies showed results in the same direction, which was defined according to significance (*p* < 0.050) [21].

### Meta-analysis

According to the Cochrane Handbook for Systematic Reviews of Interventions [22], statistically combining the results of individual studies is applicable for two or more separate studies investigating the same effect of interest. Meta-analysis will focus solely on the primary outcome measure (blood loss), whenever applicable. The *I*^2^ statistic will be reported with its 95% CI. According to von Hippel et al. [23] bias in the* I*^2^ statistic is inevitable in small meta-analyses, reporting the 95% CI along with the estimator is recommended. The random-effects model described in Chapter 10 of the Cochrane Handbook of Systematic Reviews of Interventions [22] will be applied because it allows heterogeneity between the separate studies to occur.

## Results

### Study selection

**Fig. 1:**
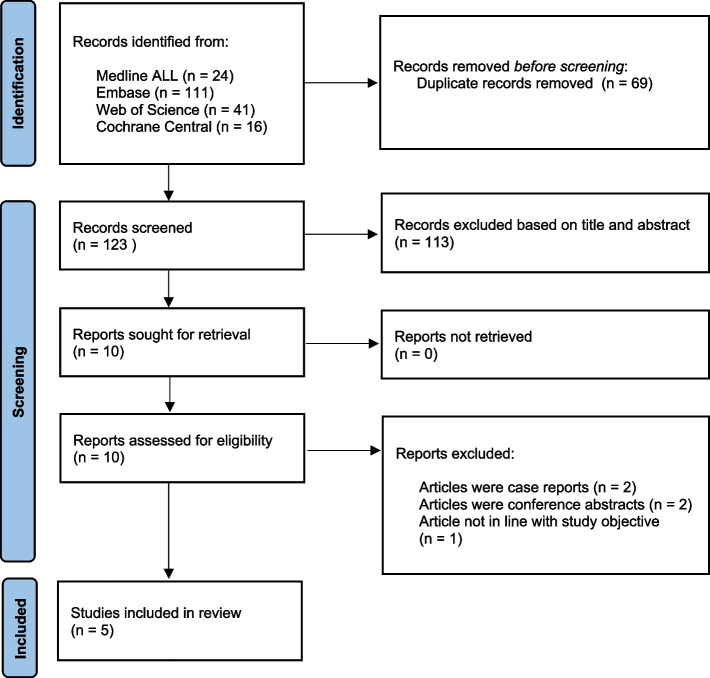
CONSORT Flow diagram of the database search

Initial literature search identified 192 articles as potentially relevant. 69 duplicates were removed which left 123 articles. Of these, 113 were excluded, due to unrelated research questions, study type or participants included in the study. Ten full text articles were thoroughly screened for eligibility. Five articles were found suitable for this systematic review. Selection of these five articles was discussed by JS and RG, consensus was reached on the inclusion of these five articles. Two articles were selected to perform a meta-analysis concerning the primary outcome (blood loss). The search strategy and selection procedure flow chart can be found in Fig. 1.

### Study characteristics & outcome measures

Five studies were included comprising two RCTs [14, 15], one prospective observational cohort study [24] and two retrospective cohort studies [11, 12]. Sample sizes of the single centre studies ranged from 30 participants [14] to 107 participants [11]. Mean %TBSA ranged from 26.72% [14] to 41.80% [15]. One study investigated the effect of tranexamic acid when administered topically [24]. The remaining four studies investigated the effect of tranexamic acid when administered intravenously. All studies included compared the administration of tranexamic acid to saline or standard of care. Blood loss was reported in three out of five studies, transfused pRBC’s in four out of five studies and Haemoglobin in three out of five studies. Table 1 shows the detailed study characteristics and Supporting Table S1 shows the study outcome measures.

**Table 1:** Characteristics of studies included

Author	Year	*N*	Study design	Intervention specification		Blood conserving strategies	Mean TBSA%		Primary outcome (Intervention vs. Control)
				**Intervention**	**Control**		**Intervention**	**Control**	
Ajai et al	2022	30	Double blind RCT	TXA 15mg/kg bolus IV	Standard of care	Adrenalin^a^ Diathermy	28.5	26.7	Less blood loss ml/cm^2^ *
Bhatia et al	2017	50	Double blind RCT	TXA 15mg/kg bolus IV	Standard of care	Adrenalin^a^ Diathermy Limb elevation	38.6	41.8	Less blood loss ml/TBSA%* Less pRBCs transfused* Higher postoperative hemoglobin level*
Mohan et al	2021	38	Prospective observational study	Topical solution adrenalin/tranexamic acid	Topical adrenalin	ND	31.0	31.0	Less blood loss ml/cm^2^ *
Tapking et al	2022	78	Retrospective case-controlled trial	TXA 10mg/kg bolus + 1mg/kg/h continuously IV	Standard of care	ND	33.5	38.5	Less blood component transfusions^a^
Domínguez et al	2017	107	Retrospective cohort study	TXA 10mg/kg bolus + 1mg/kg/h continuously IV	Historical cohort	Adrenalin^b^ Diathermy	32.8	32.9	Less pRBCs transfused*

* Statistically significant (*P*<0.05)

^a^Adrenaline soaked gauzes with the dilution 1:200.000

^b^Adrenaline soaked gauzes with the dilatation 1:500.000

*TBSA* total body surface area, *TXA* Tranexamic Acid, *pRBCs* packed Red Blood Cells, *RCT* Randomized controlled trial

### Risk of bias

**Fig. 2:**
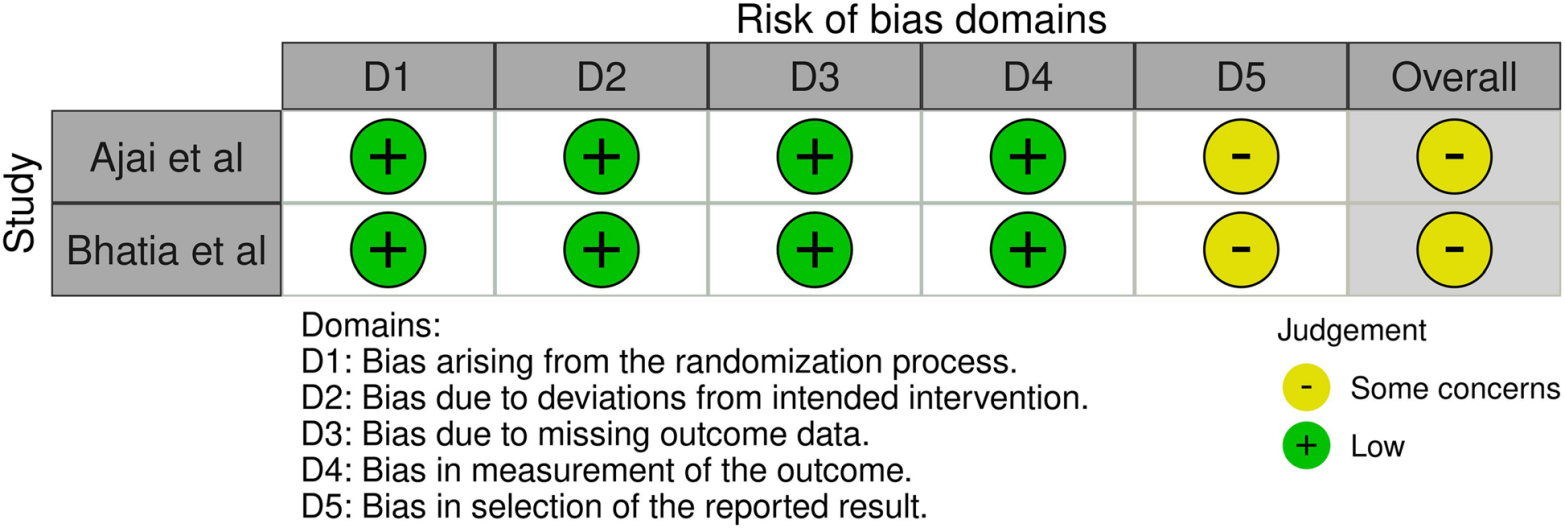
Risk of bias in randomised trials considering all outcomes

Quality assessment of the two RCTs was done with the RoB2 tool [14, 15]. These studies scored some concerns [14, 15] (see Fig. 2). Complete RoB2 assessments can be found in Multimedia Appendix A.

**Fig. 3:**
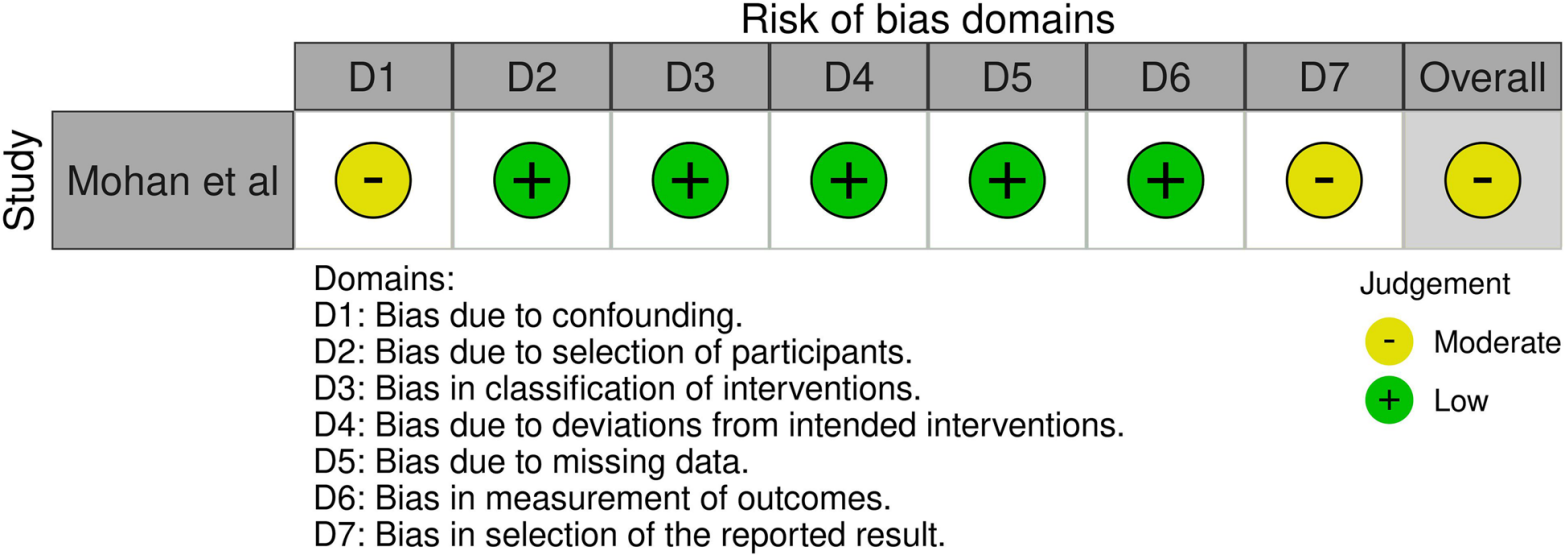
Risk of bias in Non-randomised Studies – of interventions considering outcome A Blood Loss

**Fig. 4:**
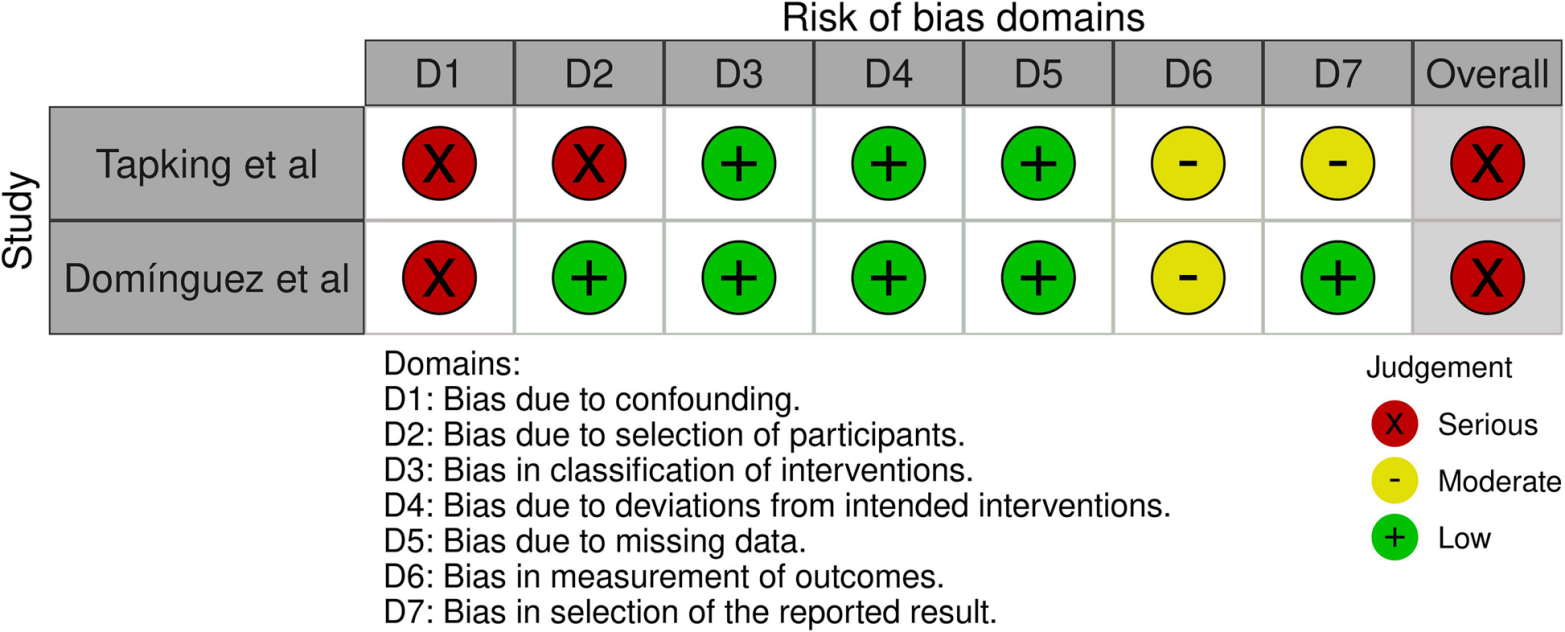
Risk of bias in Non-randomised Studies – of interventions considering outcome B transfused pRBC’s (packed Red Blood Cells)

**Fig. 5:**
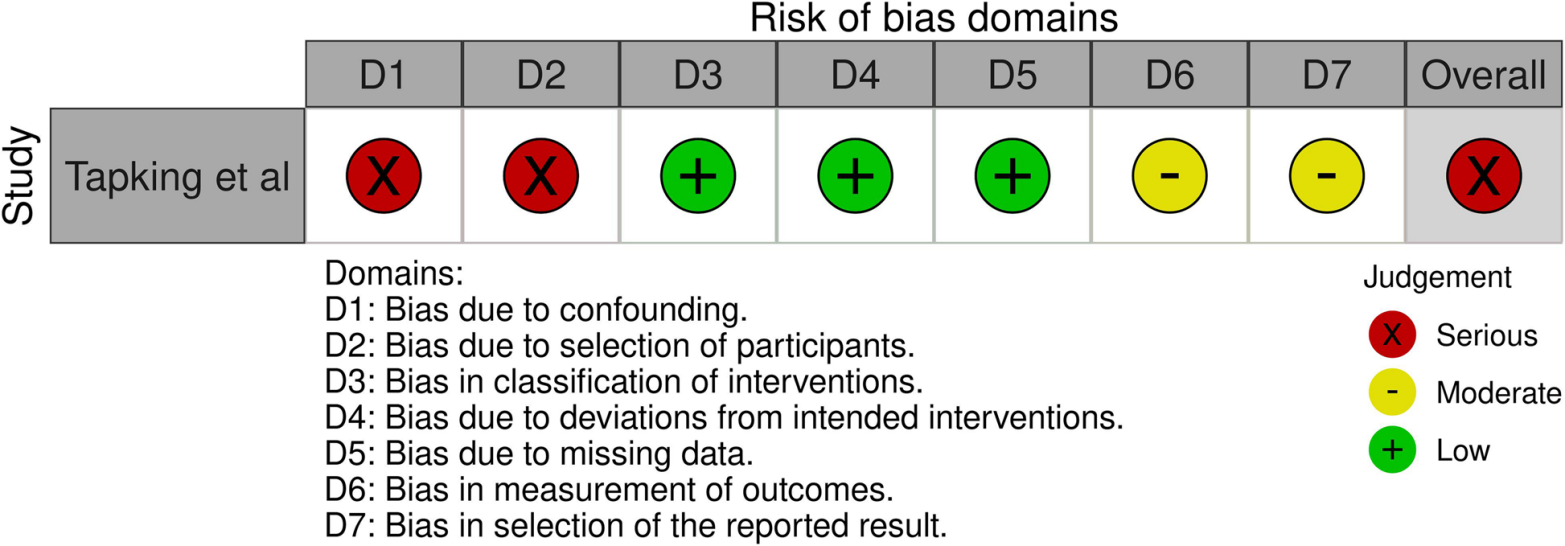
Risk of bias in Non-randomised Studies – of interventions considering outcome C Haemoglobin

Three cohort studies were included for the quality assessment with the ROBINS-I tool [11, 12, 24]. One study scored moderate risk of bias in outcome A(blood loss) [24]. Two studies scored serious risk of bias for the other outcomes B(transfused pRBC’s) and C(Haemoglobin levels) [11, 12]. Figures 3, 4 and 5 demonstrate the summarized quality assessments with the ROBINS-I tool specified per outcome A, B and C. Complete ROBINS-I assessments can be found in Multimedia Appendix B. Overall, all studies were low to moderate quality.

### Results of individual studies

Two RCTs investigated the effectiveness of tranexamic acid during surgical burn procedures. Per unit excised area there was a statistically significant reduction of blood loss in both studies: 0.45 vs. 0.73 ml/cm^2^ [14] and 14.81 vs. 23.78 ml/%TBSA [15] for tranexamic acid groups. Both RCTs also studied the transfused pRBC’s and postoperative haemoglobin levels. Only one RCT showed a statistically significant reduction of transfused pRBC’s and higher postoperative haemoglobin levels in the tranexamic acid group [15]. The other RCT failed to identify a significant reduction of transfused pRBC’s or higher postoperative haemoglobin level in the tranexamic acid group [14].

Three cohort studies reported on the effectiveness of tranexamic acid in burn surgery. One study assessed blood loss and showed a statistically significant reduction of blood loss per unit excised area in the tranexamic acid group, 1.30 vs. 2.03 ml/cm^2^ [24], when tranexamic acid was administered topically. The other two studies described statistically significant less pRBC’s transfusions in tranexamic acid groups [11, 12]. One study assessed haemoglobin levels but did not show any difference between groups. A complete overview of the results can be found in Supporting information Table S1.

### Synthesis of results

The studies included in this systematic review have heterogeneous results. The two RCTs included showed a reduction of blood loss per unit excised area in the tranexamic acid groups [14, 15]. Both RCTs investigated transfused pRBC’s [14, 15]. A reduction of transfused pRBC’s in the tranexamic acid group was found in one RCT [15]. In this RCT a higher postoperative haemoglobin level was also found in the tranexamic acid group [15].

Out of three non-RCT studies [11, 12, 24], one study investigated blood loss per unit excised area, which showed a reduction in the tranexamic acid group [24]. Two non-RCT studies investigated transfused pRBC’s, in tranexamic acid groups a reduction of transfused units was observed [11, 12]. One non-RCT investigated postoperative haemoglobin levels, the outcome was comparable for tranexamic acid and control groups accordingly [12].

All studies were of moderate or low quality. According to Proper et al. [21], in the absence of high-quality studies, multiple low-quality studies account for moderate evidence. In three studies of moderate quality the administration of tranexamic acid resulted in a reduction of blood loss per unit excised area [14, 15, 24], accounting for moderate level of evidence. In two low-quality studies and one moderate quality study the administration of tranexamic acid resulted in a reduction of transfused pRBC’s [11, 12, 15], accounting for moderate level of evidence. Postoperative haemoglobin levels were higher after tranexamic acid administration in one study, accounting for insufficient evidence [15]. Consequently, moderate level of evidence is available for the effectiveness of tranexamic acid to reduce blood loss per unit area excised and transfused pRBC’s during surgery in burn patients.

### Meta-analysis

**Fig. 6:**
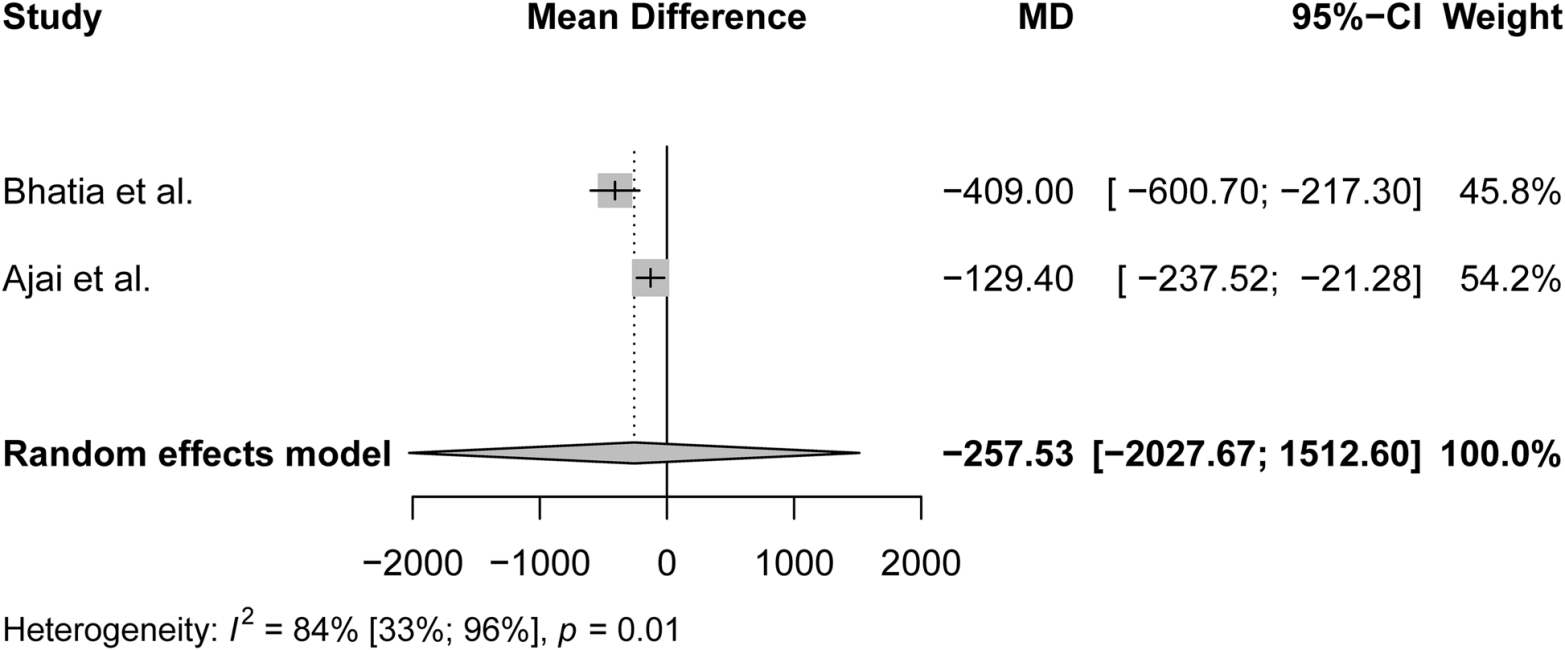
Random-effects model

The data of the two RCTs considering the overall blood loss were incorporated into a random-effects model to pool the effect of interest, since study design, patients, surgery and administration of tranexamic acid was comparable. The model failed to detect a statistically significant reduction considering the mean difference [95% CI]: -257.53 [-2027.67;1512.60]. However, substantial heterogeneity was observed. The random-effects model can be found in Fig. 6.

## Discussion

This systematic review and meta-analysis aimed to identify the effectiveness of tranexamic acid to reduce blood loss in burn patients undergoing surgery. Five studies, including two RCTs, on 303 patients, showed moderate level of evidence that tranexamic acid reduces blood loss per unit excised area and transfusion rates of pRBC’s. Postoperative haemoglobin levels were higher after tranexamic acid administration in one study, accounting for insufficient evidence. Meta-analysis on the included RCTs failed to detect an overall reduction of blood loss. Substantial heterogeneity was observed which is common if the number of studies included is small [22]. Therefore, the results from this meta-analysis should be interpreted with caution.

The effectiveness of tranexamic acid to reduce blood loss or transfusion rates is a comprehensively studied subject among a wide variety of surgical subspecialties. A meta-analysis was conducted in 2021 including 57 randomised controlled trials, in orthopaedic surgery, obstetrics and gynaecology, oral maxillofacial surgery/otolaryngology, cardiac surgery and plastic surgery [10]. Blood loss was significantly lower in the tranexamic acid groups. Standardized mean difference was -153.33 ml in patients receiving tranexamic acid [10]. Transfusion rates were significantly lower as well. Among the patients receiving tranexamic acid, a 72% reduced odds was detected [10].

Aside from meta-analyses on blood loss and transfusion requirements, the authors performed a meta-analysis on dosing of tranexamic acid as well [10]. The authors specifically focused on the dosing of tranexamic acid intravenously and these doses ranged from 10 mg/kg up to 100 mg/kg [10]. Thirty-eight studies described the dosing of tranexamic acid in their manuscripts. Twenty-one studies used a dose of 10 mg/kg for subjects, 12 studies used a dose of 15 mg/kg and 5 studies used a dose of 20 mg/kg. There was an overall trend towards less blood loss and lower odds of transfusion whenever the dose increased [10]. However, this trend was non-significant. Although this meta-analysis managed to include 57 RCTs, only one study involved burn patients [15], which was considered as plastic surgery in this meta-analysis [10]. In addition to the dosing of tranexamic acid, route of administration is described to influence the effectiveness of tranexamic acid as well.

Tranexamic acid can be administrated intravenously, but studies also describe administration by mouth, topically, or even endobronchially, according to the clinical indication [25]. Recently, studies have been published investigating a combined approach of tranexamic acid administration (topical and intravenous). A meta-analysis investigating a combined approach (topical and intravenous) of tranexamic acid in orthopaedic patients described statistically significant less blood loss and lower transfusion rates in the combined groups when compared to the single-route approaches [26]. VTE events were investigated in both groups and did not result in statistically significant differences [26]. Most research considering the use of tranexamic acid is conducted in orthopaedics, obstetrics and cardiac surgery. Dosing does not seem to influence the effect of tranexamic acid [10]. The discussion on how to administrate tranexamic acid and combining these approached has been presented among different specialties. Administrating tranexamic acid topically in addition to intravenous use seems to be beneficial in orthopaedics [26]. In burn care, studies are scarce and combined approaches are not investigated yet. Compared with specialties like orthopaedics, obstetrics or cardiac surgery there are several factors which seem to be applicable for burn patients specifically.

Some studies observed abnormal fibrinolytic function in trauma patients [27, 28] and burn patients [29]. These studies also warrant further research into the fibrinolytic function of the burn population. Trauma and burn patients might not be comparable to any other surgical patients. Tranexamic acid inhibits the degradation of fibrin [2] interfering the fibrinolytic function. Thus, the effect of tranexamic acid may be different in burn patients than already reported in other populations [10]. In addition, the use of haemostatic agents like thrombin, fibrin and epinephrine solutions to mitigate blood loss are effective to reduce blood loss, but no single technique seems superior as blood conserving protocols often adopt multiple strategies [1]. Burn patients’ fibrinolytic functions and strategies to mitigate blood loss used in burn excisional surgery should be addressed in study designs thoroughly as they can potentially influence factors like blood loos, transfusion requirements or laboratory assays like haemoglobin level [1]. Use of these strategies influence generalizability of individual study results and the inter-study comparison. But the effectiveness of tranexamic acid in individual randomized studies won’t be effected since these techniques will be used in both the intervention as the control group.

Recently, Fijany et al. [16] published a systematic review and meta-analysis considering the effectiveness of tranexamic acid in burn surgery patients. Conclusions were in contrast with conclusions from this current study. According to Fijany et al. overall blood loss significantly reduced in tranexamic acid groups from the random-effects model [16]. However, data from RCT’s and non-RCT’s were pooled in the meta-analysis. Pooling results from studies with contrasting study designs in a meta-analysis can be debated as many influential factors are balanced across study groups in RCT’s, which is in contrast with non-RCT studies. In our meta-analysis we decided to pool the results from the two RCT’s solely [14, 15]. The decision to not include the non-randomised study [24] along with the randomised studies [14, 15] can be debated, while the design between the two groups can be considered identical (within patient group design). However, the type of administration of tranexamic acid was not comparable for the non-randomised study (topical administration) versus the RCT’s (intravenous administration) [14, 15, 24], which could be associated with the outcome. Eventually, the random-effects model including two RCT’s did not provide a statistically significant reduction of overall blood loss. When corrected for the heterogeneity between the studies in the random-effects model, the mean difference’s corresponding 95% CI increased. This was probably due to the lack of evidence in the random-effect model considering the overall reduction of blood loss. Pooled results considering the transfused pRBC’s and haemoglobin levels were not generated. This was not done because that the outcome data were measured differently. For instance, transfused pRBC’s was measured in units [12] or in proportions [11] and the outcome between study groups appeared to be 0 in one study [14]. Moreover, haemoglobin levels were assessed on different moments counting from the surgery namely, direct postoperatively and 24 h postoperatively. Evidence regarding the adverse events in patients receiving tranexamic acid undergoing general surgery is growing. In a large study investigating the incidence in venous thromboembolic events between tranexamic acid and control groups in patients with traumatic brain injury was reported comparable [8]. Although this review included studies which reported no adverse events or side-effects of tranexamic acid [11, 12, 14, 15, 24], study samples were small and not powered for these outcomes.

Our systematic review and meta-analysis has several strengths. It was conducted according to the PRISMA guidelines [17] which ensured the robustness of the process. This was strengthened by the tailored search strings per electronic database, generated by the information specialist from the Erasmus MC Medical Library. This specialist has significant experience in methodological considerations concerning systematic reviews and meta-analyses. The use of separate quality assessment tools, namely the RoB2 and ROBINS-I [19, 20], specifically developed for studies with corresponding study design, enhanced the strength of the review.

However, our systematic review and meta-analysis also has some limitations. The meta-analysis was conducted based on two studies. The Cochrane Group supports the decision to perform a meta-analysis whenever two or more separate studies with comparable types of outcome data are present [22]. However, conducting a meta-analysis with a small number of studies results in unavoidable heterogeneity between studies, which can be seen in the *I*^2^ statistic [23]. Therefore, the* I*^2^ 95% CI was reported along with the estimator, as advised by von Hippel et al. [23].

Despite the above mentioned limitations, we performed a valid systematic review and meta-analysis studying the effectiveness of tranexamic acid in burn patients. Although it seems to be beneficial, the available evidence is still limited. In other specialties like orthopaedics, obstetrics and cardiac surgery evidence considering the effectiveness and dosing of tranexamic acid is growing. Dosing does not seem to influence the effect of tranexamic acid, while the route of administration and especially combining these approached does [26] Therefore, more high-quality studies investigating the effect of tranexamic acid and accounting for various administration approaches in burn patients are needed among multiple centres to retrieve conclusive results. Until then, the use of tranexamic acid in burn patients may be considered as additional therapy during a surgical procedure.

## Conclusions

This systematic review demonstrates that tranexamic acid has favourable outcomes in burn patients undergoing burn excisional surgery. Results from this systematic review and meta-analysis are heterogeneous. Moderate level of evidence is available for tranexamic acid administration to be associated with a statistically significant reduction of blood loss per unit excised area and transfused pRBC’s. Meta-analysis pooling the overall blood loss from two separate RCTs failed to detect a statistically significant reduction. Results from this meta-analysis should be interpreted with caution, while only two studies have been included in the meta-analysis. For the systematic review exclusively, there is moderate evidence available for the effectiveness of tranexamic acid in burn patients. Future research is needed. Studies should focus on the dosing of tranexamic acid, the administration approaches, and even consider combining these approaches. Future studies should also incorporate multiple study sites with larger sample sizes, to investigate the effectiveness of tranexamic acid and report on VTE events or other complications in burn excisional surgery.

### Supplementary Information

**Supplementary Material 1.**

**Supplementary Material 2.**

## Data Availability

The datasets used and/or analyzed during the current study are available from the corresponding author on reasonable request.

## Appendix

### Search strings

**Medline**

(Tranexamic Acid / OR (4-amino-methylcyclohexane-carboxyl* OR 4-aminomethylcyclohexanecarbon* OR 4-aminomethylcyclohexanecarboxyl* OR amca OR AMCHA OR amchafibrin* OR amikapron* OR aminomethyl-cyclohexane-carboxyl* OR aminomethyl-cyclohexanecarboxyl* OR aminomethylcyclohexane-carbon* OR aminomethylcyclohexane-carboxyl* OR aminomethylcyclohexanecarbon* OR aminomethylcyclohexanecarboxyl* OR aminomethylcyclohexanocarboxyl* OR aminomethylcyclohexano* OR amstat* OR anexan* OR antivoff* OR anvitoff* OR caprilon* OR cis-4-aminomethylcyclohexanecarboxyl* OR cis-aminomethyl-cyclohexanecarboxyl* OR cl-65336 OR cl65336 OR cyclocapron* OR cyclokapron* OR cyklocapron* OR cyklokapron* OR exacyl* OR fibrinon* OR frenolyse* OR hemostan* OR hexacapron* OR hexakapron* OR kalnex* OR lb-1148 OR lb114 OR lysteda* OR micranex* OR para-aminomethylcyclohexane-carboxyl* OR rikaparin* OR ronex* OR rp-18429 OR rp18429 OR theranex* OR tramic OR tranex OR tranexam* OR tranexan OR tranexic OR trans-aminomethyl-cyclohexane-carboxyl* OR trans-aminomethylcyclohexane-carboxyl* OR trans-aminomethylcyclohexanecarboxyl* OR transamin OR transaminomethylcyclohexane-carboxyl* OR transexam OR traxamic OR trenaxin OR ugurol* OR xp-12b OR xp12b).ab,ti.) AND (exp Burns / OR Burn Units / OR (burn OR burns OR burned).ab,ti.)

**Embase**

('tranexamic acid'/de OR (4-amino-methylcyclohexane-carboxyl* OR 4-aminomethylcyclohexanecarbon* OR 4-aminomethylcyclohexanecarboxyl* OR amca OR AMCHA OR amchafibrin* OR amikapron* OR aminomethyl-cyclohexane-carboxyl* OR aminomethyl-cyclohexanecarboxyl* OR aminomethylcyclohexane-carbon* OR aminomethylcyclohexane-carboxyl* OR aminomethylcyclohexanecarbon* OR aminomethylcyclohexanecarboxyl* OR aminomethylcyclohexanocarboxyl* OR aminomethylcyclohexano* OR amstat* OR anexan* OR antivoff* OR anvitoff* OR caprilon* OR cis-4-aminomethylcyclohexanecarboxyl* OR cis-aminomethyl-cyclohexanecarboxyl* OR cl-65336 OR cl65336 OR cyclocapron* OR cyclokapron* OR cyklocapron* OR cyklokapron* OR exacyl* OR fibrinon* OR frenolyse* OR hemostan* OR hexacapron* OR hexakapron* OR kalnex* OR lb-1148 OR lb114 OR lysteda* OR micranex* OR para-aminomethylcyclohexane-carboxyl* OR rikaparin* OR ronex* OR rp-18429 OR rp18429 OR theranex* OR tramic OR tranex OR tranexam* OR tranexan OR tranexic OR trans-aminomethyl-cyclohexane-carboxyl* OR trans-aminomethylcyclohexane-carboxyl* OR trans-aminomethylcyclohexanecarboxyl* OR transamin OR transaminomethylcyclohexane-carboxyl* OR transexam OR traxamic OR trenaxin OR ugurol* OR xp-12b OR xp12b):ab,ti) AND (burn/exp OR 'caustic burn'/de OR 'chemical burn'/de OR 'burn patient'/de OR 'burn care hospital'/exp OR 'burn dressing'/de OR (burn OR burns OR burned):Ab,ti)

**Web of Science**

TS=(((4-amino-methylcyclohexane-carboxyl* OR 4-aminomethylcyclohexanecarbon* OR 4-aminomethylcyclohexanecarboxyl* OR amca OR AMCHA OR amchafibrin* OR amikapron* OR aminomethyl-cyclohexane-carboxyl* OR aminomethyl-cyclohexanecarboxyl* OR aminomethylcyclohexane-carbon* OR aminomethylcyclohexane-carboxyl* OR aminomethylcyclohexanecarbon* OR aminomethylcyclohexanecarboxyl* OR aminomethylcyclohexanocarboxyl* OR aminomethylcyclohexano* OR amstat* OR anexan* OR antivoff* OR anvitoff* OR caprilon* OR cis-4-aminomethylcyclohexanecarboxyl* OR cis-aminomethyl-cyclohexanecarboxyl* OR cl-65336 OR cl65336 OR cyclocapron* OR cyclokapron* OR cyklocapron* OR cyklokapron* OR exacyl* OR fibrinon* OR frenolyse* OR hemostan* OR hexacapron* OR hexakapron* OR kalnex* OR lb-1148 OR lb114 OR lysteda* OR micranex* OR para-aminomethylcyclohexane-carboxyl* OR rikaparin* OR ronex* OR rp-18429 OR rp18429 OR theranex* OR tramic OR tranex OR tranexam* OR tranexan OR tranexic OR trans-aminomethyl-cyclohexane-carboxyl* OR trans-aminomethylcyclohexane-carboxyl* OR trans-aminomethylcyclohexanecarboxyl* OR transamin OR transaminomethylcyclohexane-carboxyl* OR transexam OR traxamic OR trenaxin OR ugurol* OR xp-12b OR xp12b)) AND ((burn OR burns OR burned)))

**Cochrane Central**

((4 NEXT amino NEXT methylcyclohexane NEXT carboxyl* OR 4 NEXT aminomethylcyclohexanecarbon* OR 4 NEXT aminomethylcyclohexanecarboxyl* OR amca OR AMCHA OR amchafibrin* OR amikapron* OR aminomethyl NEXT cyclohexane NEXT carboxyl* OR aminomethyl NEXT cyclohexanecarboxyl* OR aminomethylcyclohexane NEXT carbon* OR aminomethylcyclohexane NEXT carboxyl* OR aminomethylcyclohexanecarbon* OR aminomethylcyclohexanecarboxyl* OR aminomethylcyclohexanocarboxyl* OR aminomethylcyclohexano* OR amstat* OR anexan* OR antivoff* OR anvitoff* OR caprilon* OR cis NEXT 4 NEXT aminomethylcyclohexanecarboxyl* OR cis NEXT aminomethyl NEXT cyclohexanecarboxyl* OR cl NEXT 65336 OR cl65336 OR cyclocapron* OR cyclokapron* OR cyklocapron* OR cyklokapron* OR exacyl* OR fibrinon* OR frenolyse* OR hemostan* OR hexacapron* OR hexakapron* OR kalnex* OR lb NEXT 1148 OR lb114 OR lysteda* OR micranex* OR para NEXT aminomethylcyclohexane NEXT carboxyl* OR rikaparin* OR ronex* OR rp NEXT 18429 OR rp18429 OR theranex* OR tramic OR tranex OR tranexam* OR tranexan OR tranexic OR trans NEXT aminomethyl NEXT cyclohexane NEXT carboxyl* OR trans NEXT aminomethylcyclohexane NEXT carboxyl* OR trans NEXT aminomethylcyclohexanecarboxyl* OR transamin OR transaminomethylcyclohexane NEXT carboxyl* OR transexam OR traxamic OR trenaxin OR ugurol* OR xp NEXT 12b OR xp12b):ab,ti) AND ((burn OR burns OR burned):Ab,ti

## Abbreviations

RCTs: Randomised controlled trials
pRBC’s: Packed Red Blood Cells
PRISMA: Preferred reporting items for systematic reviews and meta-analysis
%TBSA: Percentage total body surface area
RoB2: Revised Cochrane risk of bias 2 tool for randomised controlled trials
ROBINS-I: Risk of bias tool to assess non-randomised studies of interventions
