# Supplementary material for: Effectiveness of tranexamic acid in burn patients undergoing surgery – a systematic review and meta-analysis
Source: BMC Anesthesiol. 2024 Mar 4;24:91. doi: 10.1186/s12871-024-02471-3 (PMC10910692; doi:10.1186/s12871-024-02471-3)
Supplement: Supplementary file 1 — Supplementary Material 1. [file 12871_2024_2471_MOESM1_ESM.zip › Multimedia_Appendix_1_ROB2_TXA_Burns_JS.pdf]

	A	B	C	D	E	F	G	H	I
1									[illegible]
2	**Time**	**Unique ID**	**Assessor**	**Study ID**	**Reference**	**Experimental**	**Comparator**	**Outcome**	**Results**
3	2023-05-17 13.28	1_A	JS	1	Ajai 2022	TXA 15mg/kg bolu	Saline	Outcome A	Blood Loss
4	2023-05-17 13.26	1_C	JS	1	Ajai 2022	TXA 15mg/kg bolu	Saline	Outcome C	Hemoglobin
5	2023-05-17 13.26	1_B	JS	1	Ajai 2022	TXA 15mg/kg bolu	Saline	Outcome B	packed Red Blood Cells tr
6	2023-05-17 13.26	2_A	JS	2	Bhatia 2017	TXA 15mg/kg bolu	Saline	Outcome A	Blood Loss
7	2023-05-17 13.26	2_B	JS	2	Bhatia 2017	TXA 15mg/kg bolu	Saline	Outcome B	packed Red Blood Cells tr
8	2023-05-17 13.27	2_C	JS	2	Bhatia 2017	TXA 15mg/kg bolu	Saline	Outcome C	Hemoglobin

	J	K	L
1	**Basic information**		
2	**Aim**	**Effect of adhering**	**Weight**
3	assignment to intervention (the 'intention-to-treat' effect)	NA	1
4	assignment to intervention (the 'intention-to-treat' effect)	NA	1
5	assignment to intervention (the 'intention-to-treat' effect)	NA	1
6	assignment to intervention (the 'intention-to-treat' effect)	NA	1
7	assignment to intervention (the 'intention-to-treat' effect)	NA	1
8	assignment to intervention (the 'intention-to-treat' effect)	NA	1

	M	N	O	P	Q	R	S	T
1		Domain 1. Randomization process						
2	**Sources**	**1.1**	**1.2**	**Note for 1.1&1.2**	**1.3**	**Note for 1.3**	**1.0 Algorithm result**	**1.0 Assessor's Judgemen**
3	Journal article(s)	Y	Y	Included patients were randon	N	Baseline characteristics	Low	Low
4	Journal article(s)	Y	Y	Included patients were rand	N	Baseline characterist	Low	Low
5	Journal article(s)	Y	Y	Included patients were rand	N	Baseline characterist	Low	Low
6	Journal article(s)	Y	Y	Described in paragraph 2. U	PN	Table 1 presents cha	Low	Low
7	Journal article(s)	Y	Y	Described in paragraph 2. U	PN	Table 1 presents cha	Low	Low
8	Journal article(s)	Y	Y	Described in paragraph 2. U	PN	Table 1 presents cha	Low	Low

	U	V	W	X	Y	Z	AA	AB	AC	AD	AE
1											
2	**1.0 General note**	**1.0 Optional Questic**	**1.0 Note for optic**	**2.1**	**2.2**	**Note for 2.1&2.2**	**2.3**	**Note for 2.3**	**2.4**	**Note for 2.4**	**2.5**
3				PN	N	Paragraph 2.3 states that th	NA		NA		NA
4				PN	N	Paragraph 2.3 states that	NA		NA		NA
5				PN	N	Paragraph 2.3 states that the decoding into the allocated groups and analysis was done after completion of the study procedure. Both surgeon and the anesthetist were blinded until the collected data was decoded.	NA		NA		NA
6				PN	N	The contents of syringe w	NA		NA		NA
7				PN	N	The contents of syringe w	NA		NA		NA
8				PN	N	The contents of syringe w	NA		NA		NA

	AF	AG	AH	AI	AJ	AK	AL	AM	AN
1	**Domain 2. Deviations from intended interventions**								
2	**Note for 2.5**	**2.6**	**Note for 2.6**	**2.7**	**Note for 2.7**	**2.0 Algorithm result**	**2.0 Assessor's Judgement**	**2.0 Genera**	**2.0 Optional Quest**
3		Y	CONSORT flow diagr	NA		Low	Low		
4		Y	CONSORT flow di	NA		Low	Low		
5		Y	CONSORT flow di	NA		Low	Low		
6		Y	Independent samp	NA		Low	Low		
7		Y	Independent samp	NA		Low	Low		
8		Y	Independent samp	NA		Low	Low		

	AO	AP	AQ	AR	AS	AT	AU	AV	AX	AY
1		**Domain 3. Mising outcome data**								
2	**2.0 Note for optio**	**3.1**	**Note for 3.1**	**3.2**	**Note for 3.2**	**3.3**	**Note for 3.3&3.4**	**3.4**	**3.0 Algorithm result**	**3.0 Assessor's judgement**
3		NI	No information prov	N	Because of randomiz	N		NA	Low	Low
4		NI	No information pro	N	Because of randon	N		NA	Low	Low
5		NI	No information pro	N	Because of randon	N		NA	Low	Low
6		NI	No information pro	N	Because of randon	N		NA	Low	Low
7		NI	No information pro	N	Because of randon	N		NA	Low	Low
8		NI	No information pro	N	Because of randon	N		NA	Low	Low

	AZ	BA	BB	BC	BD	BE	BF	BG	BH	BI	BJ	BK
1												**Domain 4. Measurer**
2	**3.0 Gerenal notes**	**3.0 Optional Quest**	**3.0 Note for option**	**4.1**	**Note for 4.1**	**4.2**	**Note for 4.2**	**4.3**	**Note for 4.3**	**4.4**	**Note for 4.4&4.5**	**4.5**
3		NA		N	Measure of outcon	N		N	No, randomizatio	NA		NA
4		NA		N	Not applicable for	PN	Single-center trial as	NI		N	Laboratory assa	NA
5		NA		N	Units measured, (	N		N	No, randomizatic	NA		NA
6				N	Formula describe	N	Formula was used fo	N	To obtain the ou	NA		NA
7				N		N	No, standard of care	N	No, both surgeo	NA		NA
8				PN		N	Variables are genera	N		NA		NA

	BM	BN	BO	BP	BQ	BR	BS	BT	BU	BV
1	**nent of the outcome**									
2	**4.0 Algorithm result**	**4.0 Assessor's Judgement**	**4.0 General note**	**4.0 Optional Quest**	**4.0 Note for optiona**	**5.1**	**Note for 5.1**	**5.2**	**Note for 5.2**	**5.3**
3	Low	Low				NI	Not sure, protocol o	PN	Results concerning C	PN
4	Low	Low				NI	Not sure, protocol	PN	Not applicable for a	PN
5	Low	Low				NI	Not sure, protocol	N	Not applicable for t	N
6	Low	Low				NI	Not sure, protocol	PN		PN
7	Low	Low				NI	Not sure, protocol	PN		N
8	Low	Low				NI	Not sure, protocol	N	Laboratory assays	N

	BW	BX	BY	BZ	CA	CB	CC
1	Domain 5. Selection of the reported result						
2	**Note for 5.3**	**5.0 Algorithm result**	**5.0 Assessor's Judgement**	**5.0 General**	**5.0 Optional Quest**	**5.0 Note for optiona**	**Algorithm's overall Judg**
3	Independent T-test (	Some concerns	Some concerns				Some concerns
4		Some concerns	Some concerns				Some concerns
5	See description 5.2	Some concerns	Some concerns				Some concerns
6	Independent T-test	Some concerns	Some concerns				Some concerns
7		Some concerns	Some concerns				Some concerns
8		Some concerns	Some concerns				Some concerns

	CD	CE	CF	CG	CH	CI
1	Domain 6. Overall Bias					
2	Assessor's overall Judge	6.0 General Note	6.0 Optional Question	6.0 Note for optio		
3	Some concerns					
4	Some concerns					
5	Some concerns					
6	Some concerns					
7	Some concerns					
8	Some concerns					
