# Supplementary material for: Effectiveness of tranexamic acid in burn patients undergoing surgery – a systematic review and meta-analysis
Source: BMC Anesthesiol. 2024 Mar 4;24:91. doi: 10.1186/s12871-024-02471-3 (PMC10910692; doi:10.1186/s12871-024-02471-3)
Supplement: Supplementary file 1 — Supplementary Material 1. [file 12871_2024_2471_MOESM1_ESM.zip › Multimedia_Appendix_1_ROB2_TXA_Burns_RG.pdf]

	A	B	C	D	E	F	G	H	I
1									
2	**Time**	**Unique ID**	**Assessor**	**Study ID**	**Reference**	**Experimental**	**Comparator**	**Outcome**	**Results**
3	2023-07-13 20.55	Ajaj_A	RG			TXA 15 mg/kg	placebo	Blood loss	
4	2023-07-13 20.45	Ajaj_B	RG			TXA 15 mg/kg	placebo	pRCB	
5	2023-07-13 20.50	Ajaj_C	RG			TXA 15 mg/kg	placebo	Hemoglobin	
6	2023-07-13 20.54	Bhatia_A	RG			TXA 15mg/kg	placebo	Blood loss	
7	2023-07-13 20.59	Bhatia_B	RG			TXA 15mg/kg	placebo	pRCB	
8	2023-07-13 21.05	Bhatia_C	RG			TXA 15mg/kg	placebo	Hemoglobin	

	J	K	L
1	**Basic information**		
2	**Aim**	**Effect of adhering**	**Weight**
3	assignment to intervention (the 'intention-to-treat' effect)	NA	1
4	assignment to intervention (the 'intention-to-treat' effect)	NA	1
5	assignment to intervention (the 'intention-to-treat' effect)	NA	1
6	assignment to intervention (the 'intention-to-treat' effect)	NA	1
7	assignment to intervention (the 'intention-to-treat' effect)	NA	1
8	assignment to intervention (the 'intention-to-treat' effect)	NA	1

	M	N	O	P	Q	R	S	T
1		Domain 1. Randomization process						
2	**Sources**	**1.1**	**1.2**	**Note for 1.1&1.2**	**1.3**	**Note for 1.3**	**1.0 Algorithm result**	**1.0 Assessor's Judgemen**
3	Journal article(s); Non-commercial t	PY	PY		N		Low	Low
4	Journal article(s); Non-commercial t	PY	PY		N		Low	Low
5	Journal article(s); Non-commercial t	PY	PY		N		Low	Low
6	Journal article(s)	PY	PY		N		Low	Low
7	Journal article(s)	PY	PY		N		Low	Low
8	Journal article(s)	PY	PY		N		Low	Low

	U	V	W	X	Y	Z	AA	AB	AC	AD	AE
1											
2	**1.0 General note**	**1.0 Optional Questic**	**1.0 Note for optic**	**2.1**	**2.2**	**Note for 2.1&2.2**	**2.3**	**Note for 2.3**	**2.4**	**Note for 2.4**	**2.5**
3				N	N		NA		NA		NA
4				N	N		NA		NA		NA
5				N	N		NA		NA		NA
6				N	N		NA		NA		NA
7				N	N		NA		NA		NA
8				N	N		NA		NA		NA

	AF	AG	AH	AI	AJ	AK	AL	AM	AN	AO
1	**Domain 2. Deviations from intended interventions**									
2	**Note for 2.5**	**2.6**	**Note for 2.6**	**2.7**	**Note for 2.7**	**2.0 Algorithm result**	**2.0 Assessor's Judgement**	**2.0 Genera**	**2.0 Optional Quest**	**2.0 Note for optior**
3		Y		NA		Low	Low			
4		Y		NA		Low	Low			
5		Y		NA		Low	Low			
6		Y		NA		Low	Low			
7		Y		NA		Low	Low			
8		Y		NA		Low	Low			

	AP	AQ	AR	AS	AT	AU	AV	AX	AY	AZ
1	**Domain 3. Mising outcome data**									
2	**3.1**	**Note for 3.1**	**3.2**	**Note for 3.2**	**3.3**	**Note for 3.3&3.4**	**3.4**	**3.0 Algorithm result**	**3.0 Assessor's judgement**	**3.0 Gerenal notes**
3	Y		NA		NA		NA	Low	Low	
4	Y		NA		NA		NA	Low	Low	
5	Y		NA		NA		NA	Low	Low	
6	Y		NA		NA		NA	Low	Low	
7	Y		NA		NA		NA	Low	Low	
8	Y		NA		NA		NA	Low	Low	

	BA	BB	BC	BD	BE	BF	BG	BH	BI	BJ	BK	BM
1			Domain 4. Measurement of the outcome									
2	**3.0 Optional Quest**	**3.0 Note for optio**	**4.1**	**Note for 4.1**	**4.2**	**Note for 4.2**	**4.3**	**Note for 4.3**	**4.4**	**Note for 4.4&4.5**	**4.5**	**4.0 Algorithm result**
3			PN		N		N		NA		NA	Low
4			PN		N		N		NA		NA	Low
5			PN		N		N		NA		NA	Low
6			PN		N		N		NA		NA	Low
7			PN		N		N		NA		NA	Low
8			PN		N		N		NA		NA	Low

	BN	BO	BP	BQ	BR	BS	BT	BU	BV	BW
1										**Domai**
2	**4.0 Assessor's Judgement**	**4.0 General note**	**4.0 Optional Quest**	**4.0 Note for optiona**	**5.1**	**Note for 5.1**	**5.2**	**Note for 5.2**	**5.3**	**Note for 5.3**
3	Low				PN		N		N	
4	Low				PN		N		N	
5	Low				PN		N		N	
6	Low				PN		N		N	
7	Low				PN		N		N	
8	Low				PN		N		N	

	BX	BY	BZ	CA	CB	CC	CD
1	n 5. Selection of the reported result						Domain 6.
2	5.0 Algorithm result	5.0 Assessor's Judgement	5.0 Genera	5.0 Optional Quest	5.0 Note for optiona	Algorithm's overall Judg	Assessor's overall Judge
3	Some concerns	Some concerns				Some concerns	Some concerns
4	Some concerns	Some concerns				Some concerns	Some concerns
5	Some concerns	Some concerns				Some concerns	Some concerns
6	Some concerns	Some concerns				Some concerns	Some concerns
7	Some concerns	Some concerns				Some concerns	Some concerns
8	Some concerns	Some concerns				Some concerns	Some concerns

	CE	CF	CG	CH	CI
1	**Overall Bias**				
2	**6.0 General Note**	**6.0 Optional Question**	**6.0 Note for optior**		
3					
4					
5					
6					
7					
8					
