# Supplementary material for: Effectiveness of tranexamic acid in burn patients undergoing surgery – a systematic review and meta-analysis
Source: BMC Anesthesiol. 2024 Mar 4;24:91. doi: 10.1186/s12871-024-02471-3 (PMC10910692; doi:10.1186/s12871-024-02471-3)
Supplement: Supplementary file 1 — Supplementary Material 1. [file 12871_2024_2471_MOESM1_ESM.zip › Multimedia_Appendix_B_ROBINS_B_dominguez_JS.docx]

The Risk Of Bias In Non-randomized Studies – of Interventions (ROBINS-I) assessment tool

(version for cohort-type studies)

**Version 19 September 2016**


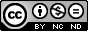


This work is licensed under a [Creative Commons Attribution-NonCommercial-NoDerivatives 4.0 International License](http://creativecommons.org/licenses/by-nc-nd/4.0/).

# ROBINS-I tool (Stage I): At protocol stage

## Specify the review question

| Participants |  |
| --- | --- |
| Experimental intervention |  |
| Comparator |  |
| Outcomes |  |

## List the confounding domains relevant to all or most studies

|  |
| --- |

## List co-interventions that could be different between intervention groups and that could impact on outcomes

|  |
| --- |

# ROBINS-I tool (Stage II): For each study

## Specify a target randomized trial specific to the study

| Design | Retrospective case-control study |
| --- | --- |
| Participants | 107 |
| Experimental intervention | TXA 10mg/kg bolus + 1mg/kg continuously IV |
| Comparator | Standard of care before introduction of TXA in unit |

## Is your aim for this study…?

| ● | to assess the effect of *assignment to* intervention |
| --- | --- |
|  | to assess the effect of *starting and adhering to* intervention |

## Specify the outcome

Specify which outcome is being assessed for risk of bias (typically from among those earmarked for the Summary of Findings table). Specify whether this is a proposed benefit or harm of intervention.

| transfused pRBC (Outcome B) |
| --- |

## Specify the numerical result being assessed

In case of multiple alternative analyses being presented, specify the numeric result (e.g. RR = 1.52 (95% CI 0.83 to 2.77) and/or a reference (e.g. to a table, figure or paragraph) that uniquely defines the result being assessed.

| 40.4 vs. 69.1% intraoperative (Intervention vs. Control)  55.8 vs. 80% perioperative (Intervention vs. Control) |
| --- |

## Preliminary consideration of confounders

Complete a row for each important confounding domain (i) listed in the review protocol; and (ii) relevant to the setting of this particular study, or which the study authors identified as potentially important.

#### “Important” confounding domains are those for which, in the context of this study, adjustment is expected to lead to a clinically important change in the estimated effect of the intervention. “Validity” refers to whether the confounding variable or variables fully measure the domain, while “reliability” refers to the precision of the measurement (more measurement error means less reliability).

| **(i) Confounding domains listed in the review protocol** | | | | |
| --- | --- | --- | --- | --- |
| Confounding domain | Measured variable(s) | Is there evidence that controlling for this variable was unnecessary?* | Is the confounding domain measured validly and reliably by this variable (or these variables)? | OPTIONAL: Is failure to adjust for this variable (alone) expected to favour the experimental intervention or the comparator? |
|  |  |  | Yes / No / No information | Favour experimental / Favour comparator / No information |
|  |  |  |  |  |
|  |  |  |  |  |
|  |  |  |  |  |
|  |  |  |  |  |
|  |  |  |  |  |
|  |  |  |  |  |
|  |  |  |  |  |
|  |  |  |  |  |

| **(ii) Additional confounding domains relevant to the setting of this particular study, or which the study authors identified as important** | | | | |
| --- | --- | --- | --- | --- |
| Confounding domain | Measured variable(s) | Is there evidence that controlling for this variable was unnecessary?* | Is the confounding domain measured validly and reliably by this variable (or these variables)? | OPTIONAL: Is failure to adjust for this variable (alone) expected to favour the experimental intervention or the comparator? |
| Demographic data | Age, gender, %TBSA burn, %TBSA full thickness, inhalation injury, use of fibrin sealant, use of intraoperative IV fluids. | Yes, because all p-values in patient characteristics between the two groups indicate that groups were comparable, which is in line with the assumption of a before and after implementation of TXA in the unit. | Yes | NA |
| Extension and depth of burn | Burn thickness, operative site, % excision | Yes, because all p-values in patient characteristics between the two groups indicate that groups were comparable, which is in line with the assumption of a before and after implementation of TXA in the unit. | Yes | NA |

* In the context of a particular study, variables can be demonstrated not to be confounders and so not included in the analysis: (a) if they are not predictive of the outcome; (b) if they are not predictive of intervention; or (c) because adjustment makes no or minimal difference to the estimated effect of the primary parameter. Note that “no statistically significant association” is not the same as “not predictive”.

## Preliminary consideration of co-interventions

Complete a row for each important co-intervention (i) listed in the review protocol; and (ii) relevant to the setting of this particular study, or which the study authors identified as important.

#### “Important” co-interventions are those for which, in the context of this study, adjustment is expected to lead to a clinically important change in the estimated effect of the intervention.

| **(i) Co-interventions listed in the review protocol** | | |
| --- | --- | --- |
| Co-intervention | Is there evidence that controlling for this co-intervention was unnecessary (e.g. because it was not administered)? | Is presence of this co-intervention likely to favour outcomes in the experimental intervention or the comparator |
|  |  | Favour experimental / Favour comparator / No information |
|  |  | Favour experimental / Favour comparator / No information |
|  |  | Favour experimental / Favour comparator / No information |
|  |  | Favour experimental / Favour comparator / No information |

| **(ii) Additional co-interventions relevant to the setting of this particular study, or which the study authors identified as important** | | |
| --- | --- | --- |
| Co-intervention | Is there evidence that controlling for this co-intervention was unnecessary (e.g. because it was not administered)? | Is presence of this co-intervention likely to favour outcomes in the experimental intervention or the comparator |
|  |  | Favour experimental / Favour comparator / No information |
|  |  | Favour experimental / Favour comparator / No information |
|  |  | Favour experimental / Favour comparator / No information |
|  |  | Favour experimental / Favour comparator / No information |

## Risk of bias assessment

Responses underlined in green are potential markers for low risk of bias, and responses in red are potential markers for a risk of bias. Where questions relate only to sign posts to other questions, no formatting is used.

|  | **Signalling questions** | **Description** | **Response options** |
| --- | --- | --- | --- |
| **Bias due to confounding** | | | |
|  | 1.1 Is there potential for confounding of the effect of intervention in this study?  **If N/PN to 1.1:** the study can be considered to be at low risk of bias due to confounding and no further signalling questions need be considered |  | PY |
|  | **If Y/PY to 1.1**: determine whether there is a need to assess time-varying confounding: |  |  |
|  | 1.2. Was the analysis based on splitting participants’ follow up time according to intervention received?  **If N/PN**, answer questions relating to baseline confounding (1.4 to 1.6)  **If Y/PY**, go to question 1.3. | Switching between groups is unlikely because this study addresses a consecutive series of patients after implementation of TXA in the unit compared to a consecutive series of patients before implementation. | N |
|  | 1.3. Were intervention discontinuations or switches likely to be related to factors that are prognostic for the outcome?  **If N/PN**, answer questions relating to baseline confounding (1.4 to 1.6)  **If Y/PY**, answer questions relating to both baseline and time-varying confounding (1.7 and 1.8) |  | NA |

|  | **Questions relating to baseline confounding only** | | |
| --- | --- | --- | --- |
|  | 1.4. Did the authors use an appropriate analysis method that controlled for all the important confounding domains? | The authors presented patient characteristics between the two groups respectively. They also describe many factors that could influence a change in outcomes measured other than the intervention. These factors are balanced between groups (statistically). Moreover, description of same surgical team and anesthetic team empowers this. | NA |
|  | 1.5. **If Y/PY to 1.4**: Were confounding domains that were controlled for measured validly and reliably by the variables available in this study? |  | NA |
|  | 1.6. Did the authors control for any post-intervention variables that could have been affected by the intervention? |  | NA |
|  | **Questions relating to baseline and time-varying confounding** | |  |
|  | 1.7. Did the authors use an appropriate analysis method that controlled for all the important confounding domains and for time-varying confounding? |  | NA |
|  | 1.8. **If Y/PY to 1.7**: Were confounding domains that were controlled for measured validly and reliably by the variables available in this study? |  | NA |
|  | **Risk of bias judgement** |  | Moderate |
|  | Optional: What is the predicted direction of bias due to confounding? |  | Unpredictable |

| **Bias in selection of participants into the study** | | | |
| --- | --- | --- | --- |
|  | 2.1. Was selection of participants into the study (or into the analysis) based on participant characteristics observed after the start of intervention?  **If N/PN to 2.1:** go to 2.4 | Yes, retrospective study | Y |
|  | 2.2. **If Y/PY to 2.1**: Were the post-intervention variables that influenced selection likely to be associated with intervention?  2.3 **If Y/PY to 2.2**: Were the post-intervention variables that influenced selection likely to be influenced by the outcome or a cause of the outcome? | Yes, characteristics described in preliminary considerations of confounders could potentially influence the selection of the intervention. However, as this is a before/after implementation study in the same unit, Table 1 presents that all these characteristics are balanced. | Y  Y |
|  | 2.4. Do start of follow-up and start of intervention coincide for most participants? | Because Length of Hospital stay is available for all included participants per group, which is presented in Table 6, one can conclude that follow up and start of intervention coincide for all participants. | Y |
|  | 2.5. **If Y/PY to 2.2 and 2.3, or N/PN to 2.4**: Were adjustment techniques used that are likely to correct for the presence of selection biases? | See description in 2.2. | NA |
|  | **Risk of bias judgement** |  | Low |
|  | Optional: What is the predicted direction of bias due to selection of participants into the study? |  | NA |

| **Bias in classification of interventions** | | | |
| --- | --- | --- | --- |
|  | 3.1 Were intervention groups clearly defined? | Yes, dosing scheme is appended. Moreover, the start and duration of intervention is also clearly described in Materials and methods sections of paper. | Y |
|  | 3.2 Was the information used to define intervention groups recorded at the start of the intervention? | Before/after implementation study, no information on when data was collected regarding the patients but length of hospital stay and presentation of burn wound characteristics assume that these factors were collected before the intervention was administrated. | PY |
|  | 3.3 Could classification of intervention status have been affected by knowledge of the outcome or risk of the outcome? | Before/after implementation study. | N |
|  | **Risk of bias judgement** |  | Low |
|  | Optional: What is the predicted direction of bias due to classification of interventions? |  | NA |

| **Bias due to deviations from intended interventions** | | | |
| --- | --- | --- | --- |
|  | **If your aim for this study is to assess the effect of assignment to intervention, answer questions 4.1 and 4.2** | |  |
|  | 4.1. Were there deviations from the intended intervention beyond what would be expected in usual practice? | There is no insight in the proportion of patients operated in total concerning the retrospective cohort. We do not know whether the anesthesiologist could have administrated TXA in the cohort before the introduction of TXA. | NI |
|  | 4.2. **If Y/PY to 4.1**: Were these deviations from intended intervention unbalanced between groups *and* likely to have affected the outcome? |  | NI |
|  | **If your aim for this study is to assess the effect of starting and adhering to intervention, answer questions 4.3 to 4.6** | |  |
|  | 4.3. Were important co-interventions balanced across intervention groups? |  | NA |
|  | 4.4. Was the intervention implemented successfully for most participants? |  | NA |
|  | 4.5. Did study participants adhere to the assigned intervention regimen? |  | NA |
|  | 4.6. **If N/PN to 4.3, 4.4 or 4.5**: Was an appropriate analysis used to estimate the effect of starting and adhering to the intervention? |  | NA |
|  | **Risk of bias judgement** |  | NI |
|  | Optional: What is the predicted direction of bias due to deviations from the intended interventions? |  | Unpredictable |

| **Bias due to missing data** | | | |
| --- | --- | --- | --- |
|  | 5.1 Were outcome data available for all, or nearly all, participants? | In the analyses number of patients are presented and all add up to 100%. | Y |
|  | 5.2 Were participants excluded due to missing data on intervention status? |  | PN |
|  | 5.3 Were participants excluded due to missing data on other variables needed for the analysis? | Authors are rigorous in presentation of all variables needed for the analysis. | PN |
|  | 5.4 **If PN/N to 5.1, or Y/PY to 5.2 or 5.3**: Are the proportion of participants and reasons for missing data similar across interventions? |  | NA |
|  | 5.5 **If PN/N to 5.1, or Y/PY to 5.2 or 5.3**: Is there evidence that results were robust to the presence of missing data? |  | NA |
|  | **Risk of bias judgement** |  | Low |
|  | Optional: What is the predicted direction of bias due to missing data? |  | NA |

| **Bias in measurement of outcomes** | | | |
| --- | --- | --- | --- |
|  | 6.1 Could the outcome measure have been influenced by knowledge of the intervention received? |  | PN |
|  | 6.2 Were outcome assessors aware of the intervention received by study participants? | Not blinded, retrospective study. | Y |
|  | 6.3 Were the methods of outcome assessment comparable across intervention groups? | Although no information is given on how the outcome assessment was collected, electronic patient records? Paper patient records? | PY |
|  | 6.4 Were any systematic errors in measurement of the outcome related to intervention received? |  | NI |
|  | **Risk of bias judgement** |  | NI |
|  | Optional: What is the predicted direction of bias due to measurement of outcomes? |  | Unpredictable |

| **Bias in selection of the reported result** | | | |
| --- | --- | --- | --- |
|  | Is the reported effect estimate likely to be selected, on the basis of the results, from... | Authors present the measurement of interest (outcome) in several ways. They also presented variables from logistic regression, whether or not statistically significant. Resulting in the fact that the authors do not report there conclusions subjectively. |  |
|  | 7.1. ... multiple outcome *measurements* within the outcome domain? |  | PN |
|  | 7.2 ... multiple *analyses* of the intervention-outcome relationship? | Presentation of outcome measurement seems appropriate, proportions of outcome variable linked to ARR’s and OR’s are valid. | PN |
|  | 7.3 ... different *subgroups*? | Authors also present outcome per subgroup in the logistic regression in which they formulated cut-off points. No information is about the motivation to determine these cut-off values. | Y |
|  | **Risk of bias judgement** |  | No information |
|  | Optional: What is the predicted direction of bias due to selection of the reported result? |  | Unpredictable |

| **Overall bias** | | | |
| --- | --- | --- | --- |
|  | **Risk of bias judgement** |  | No information |
|  | Optional: What is the overall predicted direction of bias for this outcome? |  | Unpredictable |


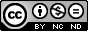


This work is licensed under a [Creative Commons Attribution-NonCommercial-NoDerivatives 4.0 International License](http://creativecommons.org/licenses/by-nc-nd/4.0/).
