# Supplementary material for: Effectiveness of tranexamic acid in burn patients undergoing surgery – a systematic review and meta-analysis
Source: BMC Anesthesiol. 2024 Mar 4;24:91. doi: 10.1186/s12871-024-02471-3 (PMC10910692; doi:10.1186/s12871-024-02471-3)
Supplement: Supplementary file 1 — Supplementary Material 1. [file 12871_2024_2471_MOESM1_ESM.zip › Multimedia_Appendix_B_ROBINS_B_JS.docx]

The Risk Of Bias In Non-randomized Studies – of Interventions (ROBINS-I) assessment tool

(version for cohort-type studies)

**Version 19 September 2016**


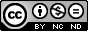


This work is licensed under a [Creative Commons Attribution-NonCommercial-NoDerivatives 4.0 International License](http://creativecommons.org/licenses/by-nc-nd/4.0/).

# ROBINS-I tool (Stage I): At protocol stage

## Specify the review question

| Participants |  |
| --- | --- |
| Experimental intervention |  |
| Comparator |  |
| Outcomes |  |

## List the confounding domains relevant to all or most studies

|  |
| --- |

## List co-interventions that could be different between intervention groups and that could impact on outcomes

|  |
| --- |

# ROBINS-I tool (Stage II): For each study

## Specify a target randomized trial specific to the study

| Design | Retrospective case-control study |
| --- | --- |
| Participants | 78 |
| Experimental intervention | TXA 10mg/kg bolus + 1mg/kg continuously IV |
| Comparator | Standard of care |

## Is your aim for this study…?

| ● | to assess the effect of *assignment to* intervention |
| --- | --- |
|  | to assess the effect of *starting and adhering to* intervention |

## Specify the outcome

Specify which outcome is being assessed for risk of bias (typically from among those earmarked for the Summary of Findings table). Specify whether this is a proposed benefit or harm of intervention.

| transfused pRBC (Outcome B) |
| --- |

## Specify the numerical result being assessed

In case of multiple alternative analyses being presented, specify the numeric result (e.g. RR = 1.52 (95% CI 0.83 to 2.77) and/or a reference (e.g. to a table, figure or paragraph) that uniquely defines the result being assessed.

| 4(3) vs. 6(4.50) units (Intervention vs. Control) |
| --- |

## Preliminary consideration of confounders

Complete a row for each important confounding domain (i) listed in the review protocol; and (ii) relevant to the setting of this particular study, or which the study authors identified as potentially important.

#### “Important” confounding domains are those for which, in the context of this study, adjustment is expected to lead to a clinically important change in the estimated effect of the intervention. “Validity” refers to whether the confounding variable or variables fully measure the domain, while “reliability” refers to the precision of the measurement (more measurement error means less reliability).

| **(i) Confounding domains listed in the review protocol** | | | | |
| --- | --- | --- | --- | --- |
| Confounding domain | Measured variable(s) | Is there evidence that controlling for this variable was unnecessary?* | Is the confounding domain measured validly and reliably by this variable (or these variables)? | OPTIONAL: Is failure to adjust for this variable (alone) expected to favour the experimental intervention or the comparator? |
|  |  |  | Yes / No / No information | Favour experimental / Favour comparator / No information |
|  |  |  |  |  |
|  |  |  |  |  |
|  |  |  |  |  |
|  |  |  |  |  |
|  |  |  |  |  |
|  |  |  |  |  |
|  |  |  |  |  |
|  |  |  |  |  |

| **(ii) Additional confounding domains relevant to the setting of this particular study, or which the study authors identified as important** | | | | |
| --- | --- | --- | --- | --- |
| Confounding domain | Measured variable(s) | Is there evidence that controlling for this variable was unnecessary?* | Is the confounding domain measured validly and reliably by this variable (or these variables)? | OPTIONAL: Is failure to adjust for this variable (alone) expected to favour the experimental intervention or the comparator? |
| Patient characteristics | TBSA (%) | No | Yes | Adjusted/matched |
| Patient characteristics | Gender | No | Yes | Adjusted/matched |
| Patient characteristics | Age | No | Yes | Adjusted/matched |
|  |  |  |  |  |
|  |  |  |  |  |
|  |  |  |  |  |
|  |  |  |  |  |
|  |  |  |  |  |
|  |  |  |  |  |
|  |  |  |  |  |

* In the context of a particular study, variables can be demonstrated not to be confounders and so not included in the analysis: (a) if they are not predictive of the outcome; (b) if they are not predictive of intervention; or (c) because adjustment makes no or minimal difference to the estimated effect of the primary parameter. Note that “no statistically significant association” is not the same as “not predictive”.

## Preliminary consideration of co-interventions

Complete a row for each important co-intervention (i) listed in the review protocol; and (ii) relevant to the setting of this particular study, or which the study authors identified as important.

#### “Important” co-interventions are those for which, in the context of this study, adjustment is expected to lead to a clinically important change in the estimated effect of the intervention.

| **(i) Co-interventions listed in the review protocol** | | |
| --- | --- | --- |
| Co-intervention | Is there evidence that controlling for this co-intervention was unnecessary (e.g. because it was not administered)? | Is presence of this co-intervention likely to favour outcomes in the experimental intervention or the comparator |
|  |  | Favour experimental / Favour comparator / No information |
|  |  | Favour experimental / Favour comparator / No information |
|  |  | Favour experimental / Favour comparator / No information |
|  |  | Favour experimental / Favour comparator / No information |

| **(ii) Additional co-interventions relevant to the setting of this particular study, or which the study authors identified as important** | | |
| --- | --- | --- |
| Co-intervention | Is there evidence that controlling for this co-intervention was unnecessary (e.g. because it was not administered)? | Is presence of this co-intervention likely to favour outcomes in the experimental intervention or the comparator |
|  |  | Favour experimental / Favour comparator / No information |
|  |  | Favour experimental / Favour comparator / No information |
|  |  | Favour experimental / Favour comparator / No information |
|  |  | Favour experimental / Favour comparator / No information |

## Risk of bias assessment

Responses underlined in green are potential markers for low risk of bias, and responses in red are potential markers for a risk of bias. Where questions relate only to sign posts to other questions, no formatting is used.

|  | **Signalling questions** | **Description** | **Response options** |
| --- | --- | --- | --- |
| **Bias due to confounding** | | | |
|  | 1.1 Is there potential for confounding of the effect of intervention in this study?  **If N/PN to 1.1:** the study can be considered to be at low risk of bias due to confounding and no further signalling questions need be considered | %TBSA could be a factor which influenced the administration of TXA for patients respectively. | PY |
|  | **If Y/PY to 1.1**: determine whether there is a need to assess time-varying confounding: |  |  |
|  | 1.2. Was the analysis based on splitting participants’ follow up time according to intervention received?  **If N/PN**, answer questions relating to baseline confounding (1.4 to 1.6)  **If Y/PY**, go to question 1.3. | Switching between intervention groups is not applicable. The decision to administrate the intervention (TXA) was taken by the anesthesiologist in charge and was applicable for the time before and during the surgery only, intervention adherence was therefore monitored. | N |
|  | 1.3. Were intervention discontinuations or switches likely to be related to factors that are prognostic for the outcome?  **If N/PN**, answer questions relating to baseline confounding (1.4 to 1.6)  **If Y/PY**, answer questions relating to both baseline and time-varying confounding (1.7 and 1.8) |  | NA |

|  | **Questions relating to baseline confounding only** | | |
| --- | --- | --- | --- |
|  | 1.4. Did the authors use an appropriate analysis method that controlled for all the important confounding domains? |  | Y |
|  | 1.5. **If Y/PY to 1.4**: Were confounding domains that were controlled for measured validly and reliably by the variables available in this study? | Presented in Table 1 with pre-matching and post-matching characteristics. Age and TBSA can be considered as objective measures. | Y |
|  | 1.6. Did the authors control for any post-intervention variables that could have been affected by the intervention? |  | N |
|  | **Questions relating to baseline and time-varying confounding** | |  |
|  | 1.7. Did the authors use an appropriate analysis method that controlled for all the important confounding domains and for time-varying confounding? | Propensity 1:2 matching. | Y |
|  | 1.8. **If Y/PY to 1.7**: Were confounding domains that were controlled for measured validly and reliably by the variables available in this study? |  | Y |
|  | **Risk of bias judgement** |  | Moderate |
|  | Optional: What is the predicted direction of bias due to confounding? |  | Unpredictable |

| **Bias in selection of participants into the study** | | | |
| --- | --- | --- | --- |
|  | 2.1. Was selection of participants into the study (or into the analysis) based on participant characteristics observed after the start of intervention?  **If N/PN to 2.1:** go to 2.4 | Yes, retrospective study. | Y |
|  | 2.2. **If Y/PY to 2.1**: Were the post-intervention variables that influenced selection likely to be associated with intervention?  2.3 **If Y/PY to 2.2**: Were the post-intervention variables that influenced selection likely to be influenced by the outcome or a cause of the outcome? | Yes, %TBSA, age, can be considered confounders for the transfusion of pRBC’s and other transfusion products. These are also related to the intervention: TXA administration.  Differences in the distribution of the primary endpoint (pRBC total) between the intervention and control group was adjusted by the van Elteren test per strata: age, sex, %TBSA. | Y  N |
|  | 2.4. Do start of follow-up and start of intervention coincide for most participants? | Because Length of Hospital stay is available for all included participants per group, which is presented in Table 1, one can conclude that follow up and start of intervention coincide for all participants. | Y |
|  | 2.5. **If Y/PY to 2.2 and 2.3, or N/PN to 2.4**: Were adjustment techniques used that are likely to correct for the presence of selection biases? | Yes, can be seen in Table 1 propensity matching of cases. | Y |
|  | **Risk of bias judgement** |  | Moderate |
|  | Optional: What is the predicted direction of bias due to selection of participants into the study? |  | Unpredictable |

| **Bias in classification of interventions** | | | |
| --- | --- | --- | --- |
|  | 3.1 Were intervention groups clearly defined? | Setting and standard of care was clearly defined in paragraph 2.1 and 2.3. Moreover, dose, frequency and intensity including the timing is presented by Figure 1, in which the dose was described concerning Ideal Body Weight of patients. | Y |
|  | 3.2 Was the information used to define intervention groups recorded at the start of the intervention? | Information used to decide whether a patient received TXA continuously or not was specified in paragraph 2.1. | Y |
|  | 3.3 Could classification of intervention status have been affected by knowledge of the outcome or risk of the outcome? | TXA has been adopted for clinical use during surgery in many specialties, A Meta-analysis and systematic review by Heyns et al, 2021 describes the use of TXA per medical specialty. This review shows the adoption for the use of TXA in medical care. Therefore, it is highly likely that the anesthesiologist who was in charge of the burn intensive care unit had the knowledge to determine to administrate TXA in patients with larger burn sizes or expected area of necrosectomy. These factors are also related to the outcome of interest pRBC. | PY |
|  | **Risk of bias judgement** |  | Low |
|  | Optional: What is the predicted direction of bias due to classification of interventions? |  | NA |

| **Bias due to deviations from intended interventions** | | | |
| --- | --- | --- | --- |
|  | **If your aim for this study is to assess the effect of assignment to intervention, answer questions 4.1 and 4.2** | |  |
|  | 4.1. Were there deviations from the intended intervention beyond what would be expected in usual practice? | Cases were selected specifically on behalf of received intervention or not. This is presented in Table 1. | N |
|  | 4.2. **If Y/PY to 4.1**: Were these deviations from intended intervention unbalanced between groups *and* likely to have affected the outcome? |  | NA |
|  | **If your aim for this study is to assess the effect of starting and adhering to intervention, answer questions 4.3 to 4.6** | |  |
|  | 4.3. Were important co-interventions balanced across intervention groups? |  | NA |
|  | 4.4. Was the intervention implemented successfully for most participants? |  | NA |
|  | 4.5. Did study participants adhere to the assigned intervention regimen? |  | NA |
|  | 4.6. **If N/PN to 4.3, 4.4 or 4.5**: Was an appropriate analysis used to estimate the effect of starting and adhering to the intervention? |  | NA |
|  | **Risk of bias judgement** |  | Low |
|  | Optional: What is the predicted direction of bias due to deviations from the intended interventions? |  | NA |

| **Bias due to missing data** | | | |
| --- | --- | --- | --- |
|  | 5.1 Were outcome data available for all, or nearly all, participants? | Table 1. Ideally an analysis plan in the appendix would be sufficient to state “Y”. | PY |
|  | 5.2 Were participants excluded due to missing data on intervention status? |  | NI |
|  | 5.3 Were participants excluded due to missing data on other variables needed for the analysis? | The intervention group was defined whether a patient received TXA or not. Whenever a patient did not receive the medication, or the staff failed to document the administration of the product, then a patient would be classified as ‘control’. Moreover, the study group was defined on behalf of 258 patients in total. But the study group consisted of 78 patients. We do not know why there were only 26 patients that received TXA. We also do not know the amount of missing data in the complete sample. It is likely that cases have been selected on behalf of completeness concerning the variables needed for the objective. | PY |
|  | 5.4 **If PN/N to 5.1, or Y/PY to 5.2 or 5.3**: Are the proportion of participants and reasons for missing data similar across interventions? |  | NI |
|  | 5.5 **If PN/N to 5.1, or Y/PY to 5.2 or 5.3**: Is there evidence that results were robust to the presence of missing data? |  | NI |
|  | **Risk of bias judgement** |  | Serious |
|  | Optional: What is the predicted direction of bias due to missing data? |  | Unpredictable |

| **Bias in measurement of outcomes** | | | |
| --- | --- | --- | --- |
|  | 6.1 Could the outcome measure have been influenced by knowledge of the intervention received? |  | N |
|  | 6.2 Were outcome assessors aware of the intervention received by study participants? | Not a blinded study, so probably yes. | PY |
|  | 6.3 Were the methods of outcome assessment comparable across intervention groups? | Fairly straight forward outcome assessment (received pRBC or not). | Y |
|  | 6.4 Were any systematic errors in measurement of the outcome related to intervention received? | It is likely that the units of transfused units in a patient is documented uniformly in all cases. Any information about the gathering of data is not included in the paper, therefore a “PN”. | PN |
|  | **Risk of bias judgement** |  | Low |
|  | Optional: What is the predicted direction of bias due to measurement of outcomes? |  | NA |

| **Bias in selection of the reported result** | | | |
| --- | --- | --- | --- |
|  | Is the reported effect estimate likely to be selected, on the basis of the results, from... |  |  |
|  | 7.1. ... multiple outcome *measurements* within the outcome domain? |  | N |
|  | 7.2 ... multiple *analyses* of the intervention-outcome relationship? |  | N |
|  | 7.3 ... different *subgroups*? | But this was appropriate to adjust for the known confounders in this study. | Y |
|  | **Risk of bias judgement** | Not an a priori statistical analysis report available. | Moderate |
|  | Optional: What is the predicted direction of bias due to selection of the reported result? |  | Unpredictable |

| **Overall bias** | | | |
| --- | --- | --- | --- |
|  | **Risk of bias judgement** |  | Serious |
|  | Optional: What is the overall predicted direction of bias for this outcome? |  | Unpredictable |


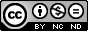


This work is licensed under a [Creative Commons Attribution-NonCommercial-NoDerivatives 4.0 International License](http://creativecommons.org/licenses/by-nc-nd/4.0/).
