# Supplementary material for: Effectiveness of tranexamic acid in burn patients undergoing surgery – a systematic review and meta-analysis
Source: BMC Anesthesiol. 2024 Mar 4;24:91. doi: 10.1186/s12871-024-02471-3 (PMC10910692; doi:10.1186/s12871-024-02471-3)
Supplement: Supplementary file 2 — Supplementary Material 2. [file 12871_2024_2471_MOESM2_ESM.pdf]

Supporting information Table S1 Study outcome measures. Values are presented as median (IQR [range]) or mean (SD).

Author	Year	Blood loss<br>Intervention vs. control	Transfused pRBC<br>Intervention vs. control	Hemoglobin<br>Intervention vs. control[ab]
Ajai et al	2022	258.7(124.1) vs. 388.1(173.9) ml<br>0.5(0.1) vs. 0.7(0.1) ml/cm² *	0.0 vs. 0.0 units	11.1(1.1) vs. 11.7(2.0) Hb-24
Bhatia et al	2017	581.0(332.2) vs. 990.0(358.9) ml *<br>14.8(7.7) vs. 23.8(8.7) ml/%TBSA *	0.3(0.5) vs. 0.8(0.9) units[a] *	8.6(1.3) vs. 7.7(1.2) Hb *<br>8.3(1.4) vs. 7.5(1.1) Hb-24 *
Mohan et al	2021	292.7(13.1) vs. 444.3(19.3) ml *<br>1.3(0.0) vs. 2.0(0.0) ml/cm² *	NR	NR
Tapking et al	2022	NR	4.0[3.0] vs. 6.0[4.5] units[b] *	10.1(1.4) vs. 9.4(1.0) Hb
Domínguez et al	2017	NR	40.4 vs. 69.1 % intraoperative pRBC transfused *<br>55.8 vs. 80.0 % perioperative pRBC transfused *	NR

Note. NR = Not Reported, * Statistically significant (P<0.050), pRBCs = packed Red Blood Cells, TBSA = total body surface area, [a] hemoglobin levels are presented as g/dL, [b] Hb-24 referring to a sample been taken 24 hours postoperatively.
